# *Leishmania amazonensis* resistance in murine macrophages: Analysis of possible mechanisms

**DOI:** 10.1371/journal.pone.0226837

**Published:** 2019-12-19

**Authors:** Sandy Santos-Pereira, Flávia O. Cardoso, Kátia S. Calabrese, Tânia Zaverucha do Valle

**Affiliations:** Laboratório de Imunomodulação e Protozoologia, Instituto Oswaldo Cruz, FIOCRUZ, Rio de Janeiro, Brazil; Universidade de Sao Paulo Instituto de Ciencias Biomedicas, BRAZIL

## Abstract

Leishmaniasis encompass a group of infectious parasitic diseases occurring in 97 endemic countries where over one billion people live in areas at risk of infection. It is in the World Health Organization list of neglected diseases and it is considered a serious public health problem, with more than 20,000 deaths a year and high morbidity. Infection by protozoa from the genus *Leishmania* can cause several forms of the disease, which may vary from a self-healing ulcer to fatal visceral infection. *Leishmania* species, as well as host immune response and genetics can modulate the course of the disease. *Leishmania* sp are obligatory intracellular parasites that have macrophages as their main host cell. Depending on the activation phenotype, these cells may have distinct roles in disease development, acting in parasite control or proliferation. Therefore, the purpose of this work was to analyze *Leishmania amazonensis* infection in primary macrophage cells obtained from mice with two distinct genetic backgrounds, ie. different susceptibility to the infection; evaluating the cause for that difference. After infection, peritoneal macrophages from the resistant C3H/He strain presented lower parasite load when compared to susceptible BALB/c macrophages. The same was also true when cells received a Th2 stimulus after infection, but the difference was abrogated under Th1 stimulus. Nitric oxide production and arginase activity was different between the strains under Th1 or Th2 stimulus, respectively, but iNOS inhibition was unable to suppress C3H/He resistance. Hydrogen peroxide production was also higher in C3H/He than BALB/c under Th1 stimulus, but it could not account for differences in susceptibility. These results led us to conclude that, although they have an important role in parasite control, neither NO nor H_2_O_2_ production can explain C3H/He resistance to infection. Other studies are needed to uncover different mechanisms of resistance/susceptibility to *L*. *amazonensis*.

## Introduction

Leishmaniasis are a group of parasitic diseases with a wide geographic distribution, which are considered a public health problem in several countries, as reported by the World Health Organization [1]. Among the affected countries, Brazil has one of the largest numbers of reported cases, according to the Pan-Americana Health Organization [2]. This set of diseases is caused by a protozoan of the genus *Leishmania*, which is transmitted to the mammalian hosts through the bite of the female sand fly [3,4]. Leishmaniasis are spectral diseases and their broad spectrum of clinical manifestations is influenced by several factors, including the species and genetics of the *Leishmania*, the interaction of the parasite with the immune response and the host genetics [5–7].

In vertebrate hosts, macrophages are the main infected cells, which are, in turn, essential for the establishment of infection and persistence of *Leishmania*. However, in addition to harboring the parasite, these cells act on parasite control and modulation of the subsequent immune response. The sustainment or elimination of the infection will, therefore, be largely dependent on the type of macrophage activation [8,9].

Macrophages are cells with high plasticity equipped with homeostatic functions. Their phenotype is influenced by the microenvironment in which it is inserted [10,11]. Upon receiving proinflammatory stimuli, such as IFN-γ and lipopolysaccharide (LPS), the macrophage is programmed into an M1 profile (classical activation). As a result of this activation, it produces high levels of reactive oxygen species (ROS), nitric oxide (NO) and pro-inflammatory cytokines such as IL-12 and TNF-α, leading to increased microbicidal capacity [12,13]. This activation is essential for the elimination of the *Leishmania* parasite [9,14]. Nevertheless, when the macrophage is stimulated by anti-inflammatory cytokines, such as IL-4 and IL-13, it acquires a M2 profile (alternative activation). This type of activation promotes the production of proline, polyamines and urea. As polyamines are nutritional supports for the *Leishmania* [8,14], the alternative activation of the macrophage favors the replication of the parasite [15].

Murine experimental models of *Leishmania* infection have been used as a tool to study immunology [16] as well as parasite resistance/susceptibility [17]. Mice from the C3H/He strain are considered resistant to infection by *L*. *amazonensis*, developing only a small, self-resolving lesion. A hundred and twenty days after infection, these animals presented no sign of injury or parasites, although parasites could still be isolated from their draining lymph node [18]. The extracellular matrix at the site of infection was also restructured [19]. For this reason, in this work we studied *ex vivo* macrophages from two distinct genetic backgrounds, with the purpose of analyzing the role of these cells in the control or growth of parasites in the initial times of *L*. *amazonensis* infection. Ultimately, this may lead to a better understanding of the mechanisms involved in the resistance of C3H/He animals.

## Methods

### Cells and parasites

*L*. *amazonensis* (MHOM/BR/2000/MS501) is maintained by successive passages in female BALB/cAn mice and periodically reisolated from the popliteal lymph node. *In vitro*, promastigote forms were maintained in axenic culture in Schneider's Insect medium (Sigma-Aldrich, St. Louis, MO) supplemented with 10% inactivated fetal bovine serum (Cultilab), 100 U/mL penicillin and 10 μg/mL streptomycin (Sigma-Aldrich, St. Louis, MO) at 26ºC in a BOD incubator, for a maximum of 6 passages. The strain, originally isolated from a human visceral case, has been characterized by isoenzymes and RFLP [20].

Peritoneal macrophages were obtained by peritoneal lavage of BALB/cAn and C3H/He mice (ICTB/FIOCRUZ) 72 hours after 3% thioglycollate intraperitoneal injection. After the lavage, cells were diluted in RPMI medium without phenol red (Sigma-Aldrich, St. Louis, MO) supplemented with 10% fetal bovine serum (Cultilab, Campinas, Brazil), 200 mM L-glutamine, 100 U/mL penicillin and 10 μg/mL streptomycin (Sigma-Aldrich, St. Louis, MO), seeded in a culture dish or over coverslips and kept at 34°C in a humidified atmosphere with 5% of CO_2_. The BALB/cAn animals will be referred to in the article as BALB/c.

### Ethics statement

Procedures with animals were performed in accordance to the Brazilian legislation on the use of animals for scientific research, as regulated by the Conselho Nacional de Controle de Experimentação Animal (CONCEA). Experiments were carried out after approval by Instituto Oswaldo Cruz (IOC) Institutional Animal Care and Use Committee (Comissão de ética no uso de animais—CEUA—IOC) under the number L-030/2016. Euthanasia was performed by anesthetic overdose (ketamin associated with xylazine) followed by cervical dislocation to ensure death.

### Infection of peritoneal macrophages

Peritoneal macrophages were infected by *L*. *amazonensis* promastigotes always at a ratio of 2 parasites per cell. Axenic cultures of promastigote forms in stationary phase were left in contact with the cells for 6 hours. After, the culture was washed with PBS pH 7.0 to remove promastigotes from the supernatant.

Experiments with pro-inflammatory stimulation used 5 μg/mL of LPS (Sigma-Aldrich, St. Louis, MO) and 2 ng/mL of recombinant mouse IFN-γ (BD Pharmingen^™^, BD Biosciences, San José, CA). In experiments with anti-inflammatory stimulation, 2 ng/mL of recombinant mouse IL-4 (BD Pharmingen^™^, BD Biosciences, San José, CA) were used. Cells were stimulated 6 hours after infection. The number of cells used in each experiment is described in S1 Table.

### Determination of the intracellular parasite load

At 24, 48, 72 and 96 hours post infection coverslips with infected macrophages were fixed and stained with Giemsa (Merck®, Darmstadt, DE). Infection rate, mean number of amastigotes/cell and total number of amastigotes/100 cells were calculated by counting 100 cells/coverslip.

### Nitric oxide (NO) production

Nitric oxide production was estimated by the Griess reaction [21]. Briefly, 50 μL of cell supernatant were added to 50 μL of Griess reagent (25 μL of 0.1% solution of N-(1-Naphthyl) ethylenediamine dihydrochloride (Sigma-Aldrich, St. Louis, MO) and 25 μL of 1% sulfanilamide solution (Fluka) in 2.5% H_3_PO_4_. After 10 minutes in the dark, samples absorbance were read at 570 nm. The amount of NaNO_2_ was calculated by comparison with a standard curve ranging from 0 to 100 μM of NaNO_2_. Non-infected cells were also essayed for control purposes.

### Hydrogen peroxide (H_2_O_2_) production

The hydrogen peroxide production was estimated by the method of Pick and Keisari [22] modified by Pick and Mizel [23]. Briefly, cell supernatant was removed and 100 μL of a solution containing 140 mM NaCl (Sigma-Aldrich, St. Louis, MO), 10 mM potassium phosphate buffer, pH 7.0, 5.5 mM dextrose (Sigma-Aldrich, St. Louis, MO), 0.56 mM (0.2 g/l) phenol red (Sigma-Aldrich, St. Louis, MO) and 0.01 mg/mL horseradish peroxidase type II (Sigma-Aldrich, St. Louis, MO) was added. Non-infected cells were used as negative control and non-infected cells treated with 0.2 μM phorbol myristate acetate (PMA) (Sigma-Aldrich, St. Louis, MO) were used as positive control. The plates were maintained for 1 hour at 37 C and 5% CO_2_, after which the reaction was stopped with 50 μL 5 N NaOH and the absorbance was read at 620 nm. A standard curve with known concentrations of H_2_O_2_ (0–100 μM) was used to determine the production of H_2_O_2_ by peritoneal macrophages.

### Arginase activity

The activity of arginase was evaluated according to Classen et al [12]. Briefly, cells were lysed in a 0.1% triton solution under agitation. Next, 100μL of a 50mM of Tris-HCl pH 7.5 solution was added to each well and samples were incubated at 56°C for 7 minutes, before addition of 0,5M of arginine (pH 9.7) and a new incubation at 37°C for 1 hour. Reaction was stopped by an acidic mix of H_3_PO_4_, H_2_SO_4_, in water (1:3:7) before addition of α-Isonitrosopropiophenone (ISPP 6% solution, Sigma-Aldrich, St. Louis, MO). After subsequent 30 minutes-incubations at 95°C and 4°C, absorbance was read at 540 nm. A standard curve with known concentrations of urea (0–180μg) was used to estimate arginase activity by peritoneal macrophages. The experiment was performed with anti-inflammatory stimulation and without stimulation, in infected and non-infected cells.

### iNOS and arginase mRNA determination

Total RNA was extracted from cells using the TRIreagent^®^ (ThermoFisher, Whaltan, MA) following the manufacturer’s instructions. cDNA synthesis was performed with 1 μg of total RNA with the iScript cDNA Synthesis kit (BD Pharmingen^™^, BD Biosciences, San José, CA) according to the manufacturer’s recommendations. The Real Time PCR assays were performed by Power SYBR^®^ Green Master Mix and the relative quantification method was applied, using the mouse gene TUBB5 as the endogenous control. For mRNA quantification, specific primers for iNOS, Arginase and Tubb5 (100nM) were used (Table 1). Reactions were conducted in a QuantStudio 3 System (Applied Biosystems, Foster City, CA). The temperature parameters consisted of a hold at 95°C for 10 min followed by 40 cycles of 95°C for 15 s and 58°C for 1 min. A melt curve analysis was performed on all reactions. The results were analyzed with the QuantStudio^™^ Design & Analysis Software (Applied Biosystems, Foster City, CA).

**Table 1:** Primers used for real time PCR.

Target	Primer sequence		Sequence source
	Forward (5’- 3’)	Reverse (5’- 3’)	
**iNOS**	GGATCTTCCCAGGCAACCA	CAATCCACAACTCGCTCCAA	NM_010927
**Arginase I**	GGTCCACCCTGACCTATGTGT	ACGATGTCTTTGGCAGATATGC	NM_007482.3
**Tubb5**	GATCGGTGCTAAGTTCTGGGA	AGGGACATACTTGCCACCTGT	NM_011655.5

### Inhibition of iNOS

100μM of 1400W dihydrochloride (Sigma-Aldrich, St. Louis, MO), a specific iNOS inhibitor [24], was added just after macrophage plating and repeated after PBS washing following infection. Infection was estimated by amastigotes counting 72 and 96h after infection, as described above.

### Statistical analysis

Each experiment was carried out three times, always in triplicate. Values were expressed as mean ± SD. Results were analyzed by Analysis of Variance (ANOVA) Sidak’s multiple comparisons test, using GraphPad Prism 7. Differences were considered significant when *p* < 0.05.

## Results

### C3H/He macrophages are less permissive to the multiplication of *L*. *amazonensis* amastigotes than BALB/c’s

**Fig 1:**
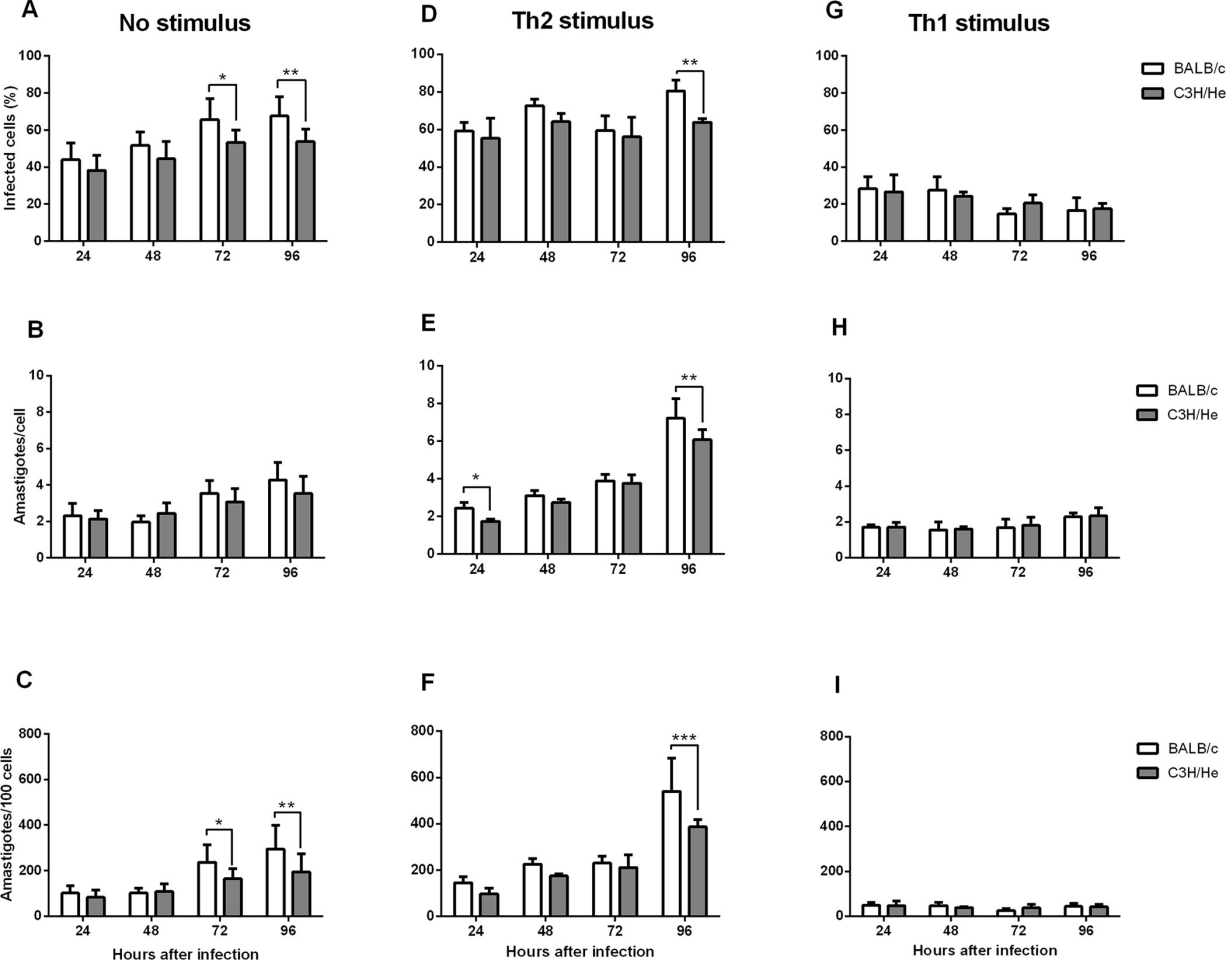
Intracellular amastigote count in BALB/c and C3H/He macrophages. Peritoneal macrophages of BALB/c and C3H/He were infected with *L*. *amazonensis* promastigotes (2 MOI). After Giemsa staining, 100 cells/coverslip were counted in order to estimate the percentage of infected cells, mean number of amastigotes per cell and total amount of amastigotes in a hundred cells at 24, 48, 72 and 96 hours after infection. (A-C) non-stimulated cells; (D-F) cells were stimulated with IL-4 (2 ng/mL) 6 hours after infection (Th2 stimulus); (G-I) cells were stimulated with IFN-γ (2 ng/mL) and LPS (5 μg/mL) 6 hours after infection (Th1 stimulus). Statistical analysis were performed by ANOVA followed by a Sidak’s multiple comparisons test. *p<*0*,*05*; **p<*0*,*01*, ***p<*0*,*001*. Bars represent mean ± SD.

Based on the difference in susceptibility previously observed between C3H/He and BALB/c mice infected with *L*. *amazonensis* [20], we hypothesized that the response to infection of the main host cell of *Leishmania* parasite, the macrophages, would be distinct, determining the resistance/susceptibility profile in C3H/He and BALB/c animals. From this premise, it was suggested that C3H/He macrophages would be able to control *L*. *amazonensis* infection. In order to verify this hypothesis, stationary phase promastigotes were used to infect cells from BALB/c and C3H/He genotypes and calculate the infection rate. At 24 hours after infection, there was no difference in the amount of infected cells from each genetic background (Fig 1A), leading to the conclusion that both cells are permissive to infection. To verify if there was a difference in the multiplicative capacity of amastigotes in peritoneal macrophages of both lines, the infection was followed for 96 hours, analyzing the quantity of amastigotes per cell. From 72h of infection, the infection rate (Fig 1A) and total number of amastigotes (Fig 1C) increased in BALB/c macrophages, but remained stable in C3H/He cells, suggesting that the later does not allow the dissemination of infection.

### Stimulation by IL-4 increases the susceptibility of both macrophage lineages

Depending on the microenvironment in which macrophages are inserted, their response may favor the survival or elimination of intracellular protozoa. BALB/c mice is considered susceptible to *L*. *major* and several other parasites because it mounts an early Th2 response [25]. Therefore, we hypothesized that, by creating an environment that favors a response pro-multiplication of amastigotes, we would make C3H/He cells as susceptible as BALB/c cells. Thus, we stimulated the cultures with IL-4 after infection and repeated the experiments of intracellular amastigote count. When the infection of IL-4-stimulated cells was compared to non-stimulated cells, a significant increase in the infection rate at 24 and 48 hours after infection, as well as at the parasite load at 48h and 96h was observed (S1A–S1C Fig). These results suggest that both strains responded to the stimulus, favoring parasite proliferation. However, IL-4-treated C3H/He macrophages showed lower infection rate, lower amount of amastigotes per cell, and lower total parasite load 96h after infection compared to IL-4-treated BALB/c macrophages (Fig 1D–1F), showing that, even under anti-inflammatory stimulus, the C3H/He macrophages remained more resistant to infection than BALB/c. In conclusion, incubation with IL-4 let C3H/He and BALB/c macrophages more susceptible to infection, but the difference in susceptibility was unaltered.

On the other hand, if the parasite load of the IL-4-stimulated C3H/He macrophages is compared to non-stimulated BALB/c macrophages, there is an inversion 48 and 96 hours after infection (S1C Fig). This shows that anti-inflammatory stimulus is capable of increasing the susceptibility of C3H/He macrophages, making it more susceptible than non-stimulated BALB/c cells.

### Pro-inflammatory stimulus equates BALB/c and C3H/He microbicidal activity

Since IL-4 was able to increase cell susceptibility, we also evaluated the response profile of macrophages under pro-inflammatory stimulus. For this, we stimulated macrophages of both strains with IFN-γ and LPS. At this condition, a reduction in total parasite load and infection rate was observed in cells from both strains when compared to non-stimulated cells, showing that IFN/LPS stimulus was able to make cells more resistant to infection (S1D–S1F Fig). However, no difference was observed either in the number of amastigotes per cell, infection rate or total parasite load between BALB/c and C3H/He stimulated-cells (Fig 1G–1I). Thereby, the pro-inflammatory stimulus was capable to equate the microbicidal activity in macrophages of both lineages, and significantly reduce the parasitic load on both of them.

### C3H/He macrophages without stimulus have similar arginase activity but higher NO production than BALB/c

**Fig 2:**
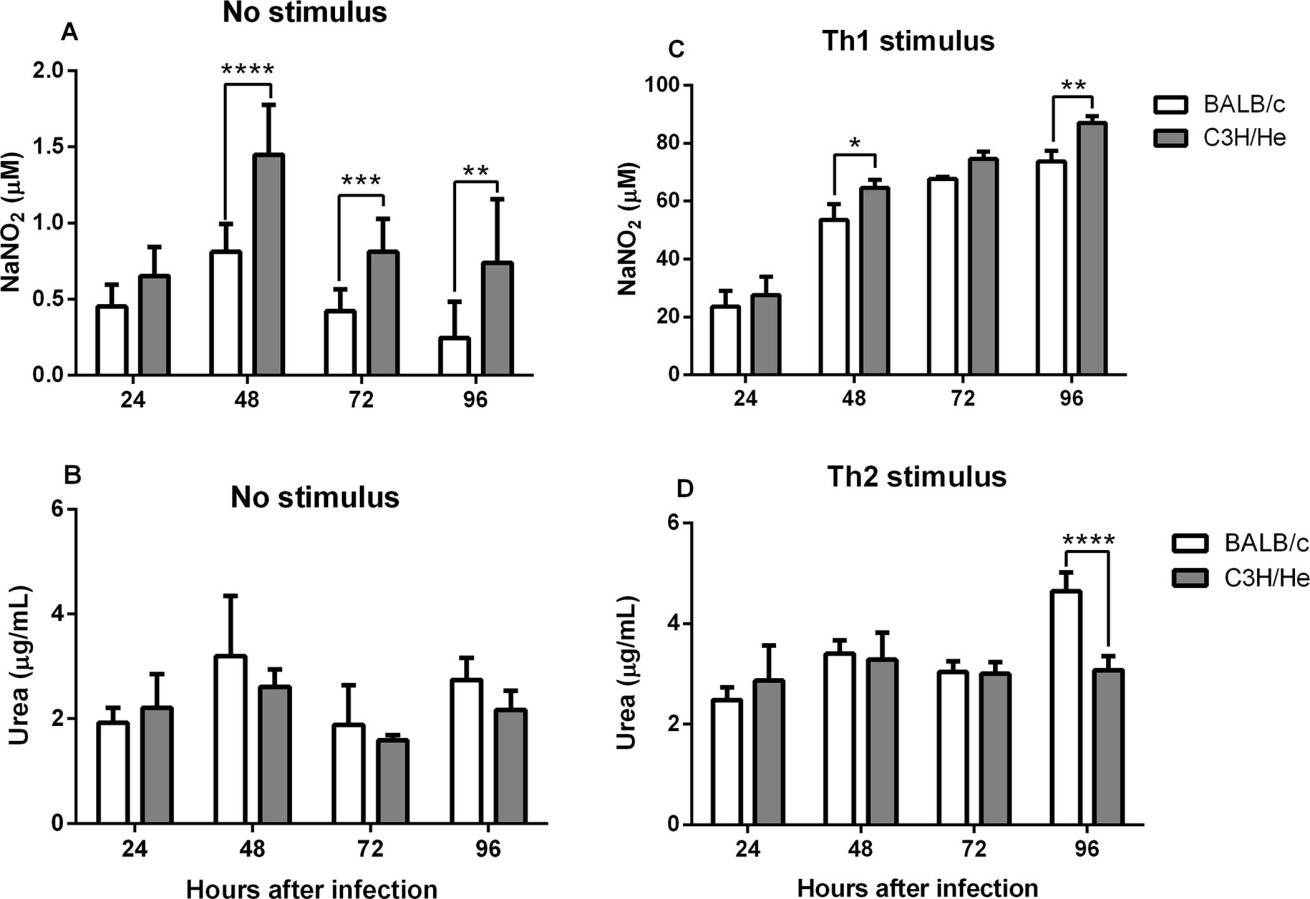
Nitric oxide production and arginase activity. (A) Peritoneal macrophages of BALB/c and C3H/He strains infected with *L*. *amazonensis* (2 MOI). Nitric oxide production (A) and arginase activity (B) in non-stimulated cells. (C) Nitric oxide production after Th1 stimulus (IFN- γ 2 ng/mL and LPS 5 μg/mL 6 hours after infection). (D) Arginase activity in Th2-stimulated cells (IL-4 2 ng/mL 6 hours after infection). Arginase activity was measured in cell lysate at different time points after infection. Nitric oxide production was measured from the cell supernatant by Griess reaction at different times after infection. Statistical analysis were performed by ANOVA followed by a Sidak’s multiple comparisons test. *p<*0*,*05*; **p<*0*,*01*, ***p<*0*,*001*, ****p<*0*,*0001*. Bars represent mean ± SD.

The microbicidal capacity of the macrophage depends largely on its oxidative metabolism, the production of NO and the arginase activity. Therefore, NO production and arginase activity were evaluated in *L*. *amazonensis*-infected macrophages from both genetic backgrounds. In unstimulated cells, Griess reaction was able to detect higher NO production in C3H/He than BALB/c infected cells, despite the fact that the supernatant of cultures of both lineages presented extremely low NaNO_2_ levels (Fig 2A). This result corroborates the difference observed between strains in infection rate and parasitic load at the same condition. When the amount of iNOS mRNA was evaluated by RT-qPCR the amount of mRNA was minimal on both cells. On the other hand, the arginase activity in unstimulated cells did not present any difference between the lineages (Fig 2B) just as the in mRNA. When cells received the Th2 stimulus, NO production on both cells was extremely low and could not be quantified, but the arginase activity of BALB/c infected macrophages was significantly higher than C3H/He’s cells 96 hours after infection (Fig 2D). This result was in accordance with the higher susceptibility of BALB/c cells and suggested that this pathway could be implicated in the susceptibility to *L*. *amazonensis*. On the other hand, when under Th1 stimulus, C3H/He presented higher NO production than BALB/c, even though the infection rate and parasite load at this condition was the same on both genetic backgrounds (Fig 2C).

### iNOS enzyme activity is not primordial for the leishmanicidal capacity of the C3H/He macrophages

**Fig 3:**
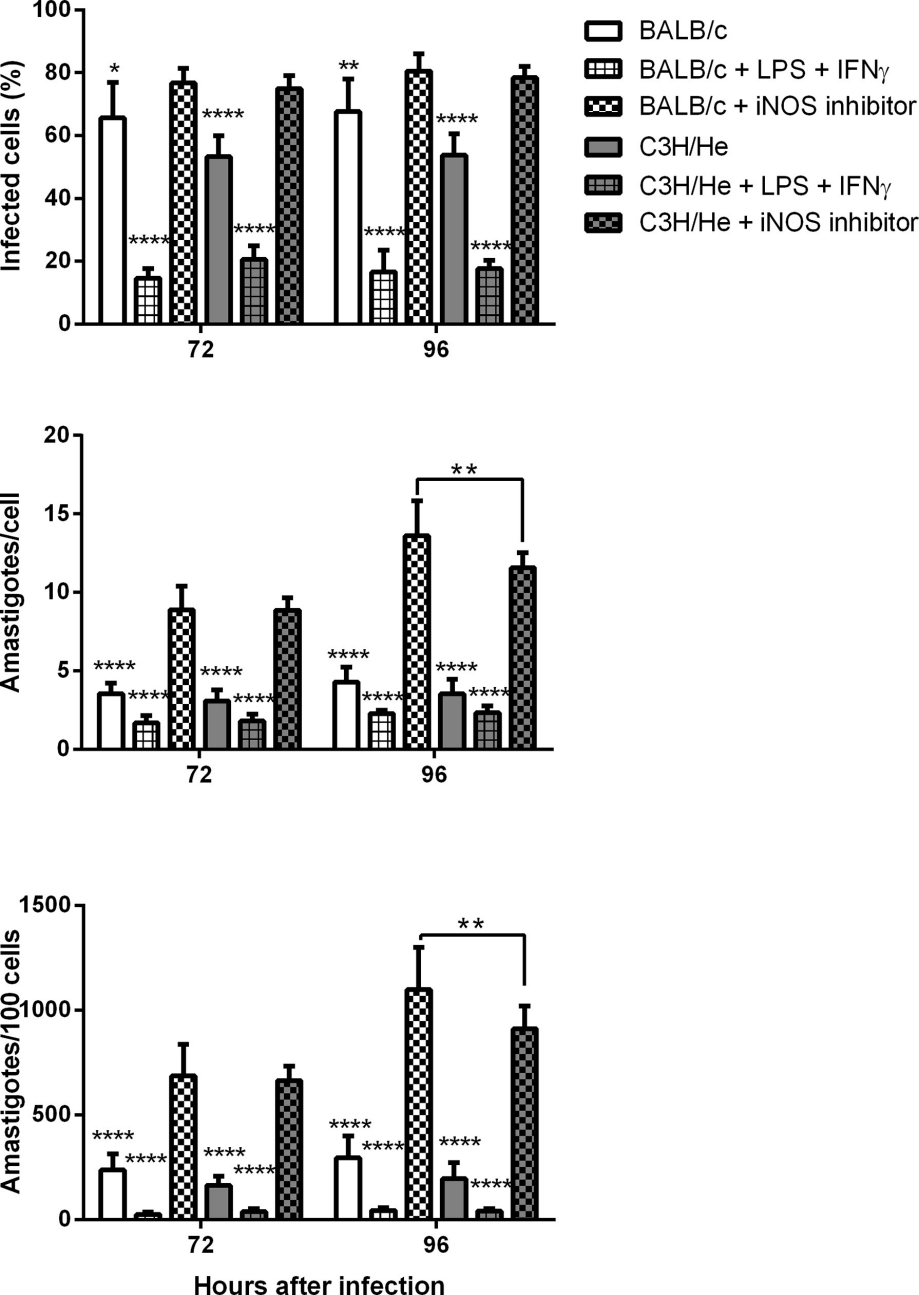
Intracellular amastigote count after iNOS inhibition. Peritoneal macrophages of BALB/c and C3H/He were treated with 100 μM of 1400W dihydrochloride after macrophage plating and infection with *L*. *amazonensis* (2 MOI). Non-treated control cells were either untreated or received IFN- γ (2 ng/mL) and LPS (5 μg/mL) 6 hours after infection. Coverslips were stained with Giemsa and intracellular amastigotes were counted in 100 cells at different times after infection. **(A)** Infection Rate; (**B)** Average number of amastigotes per cell; (**C)** Total parasitic load. Statistical analysis were performed by ANOVA followed by a Tukey’s multiple comparisons test, comparing the iNOS-inhibited group with each of the others, at the different time points, as well as comparing cells in the same condition but with distinct genetic backgrounds. *p<*0*,*05*; ****p<*0*,*0001*. Bars represent mean ± SD.

In order to undoubtedly assess whether C3H/He resistance could be dependent on increased NO production, we inhibited iNOS activity in macrophages using 1400W dihydrochloride, starting before infection, at the time of cell plating (S2 Fig). NO inhibition was able to increase the infection rate and parasite load on both BALB/c and C3H/He, but the total parasite burden was still lower on C3H/He macrophages (Fig 3). These results show that, although NO certainly participates on parasite control, it is not the main mechanism responsible for C3H/He resistance.

### Hydrogen peroxide production is higher in Th1-stimulated C3H/He macrophages

**Fig 4:**
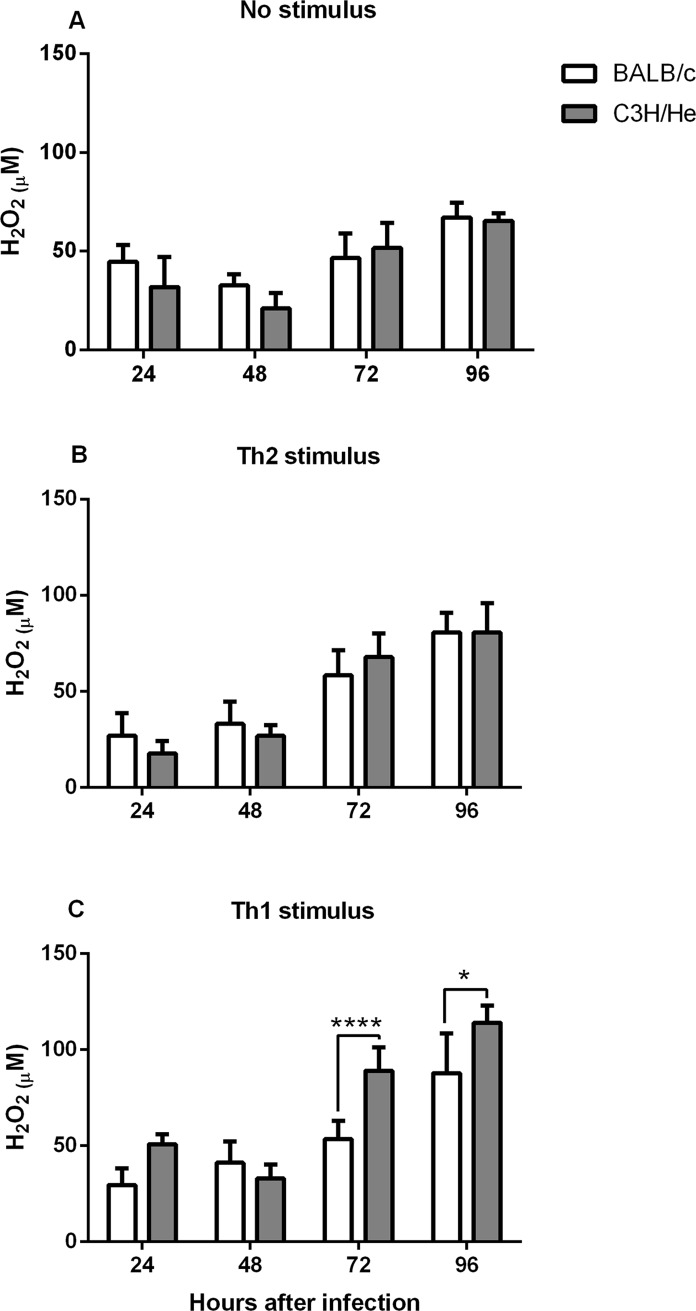
Hydrogen peroxide production. Peritoneal macrophages of BALB/c and C3H/He were infected with *L*. *amazonensis* promastigotes (2 MOI). ROS production was estimated by the measurement of H_2_O_2_ in cultures supernatants 24, 48, 72 and 96 hours after infection. (A) non-stimulated cells; (B) cells were stimulated with IL-4 (2 ng/mL) 6 hours after infection (Th2 stimulus); (C) cells were stimulated with IFN-γ (2 ng/mL) and LPS (5 μg/mL) 6 hours after infection (Th1 stimulus). Statistical analysis were performed by ANOVA followed by a Sidak’s multiple comparisons test. *p<*0*,*05*; ****p<*0*,*0001*. Bars represent mean ± SD.

In addition to NO, macrophages also produce reactive oxygen species (ROS) in response to infection. The enzyme NADPH oxidase generates superoxide radicals, which are then converted to more toxic hydrogen peroxide (H_2_O_2_) [26]. ROS has been implicated in the killing of *L*. *braziliensis* by monocytes from patients with cutaneous leishmaniasis [27]. Therefore, we hypothesized that it might be involved in C3H/He resistance. However, when we estimated H_2_O_2_ concentration in the supernatant of *L*. *amazonensis*-infected and non-stimulated macrophages, no difference was observed between BALB/c and C3H/He. The same was true for the supernatant of IL-4 stimulates cells, but when cells received a Th1 stimulus, C3H/He released more H_2_O_2_ than BALB/c (Fig 4). Although interesting, these data do not correlate with the differences observed in infection rate and parasite load, since Th1-stimulated cells from both BALB/c and C3H/He presented similar infection (Fig 1G–1I).

## Discussion

We have used the differences in susceptibility between BALB/c and C3H/He mice to search for cellular mechanisms that might influence *Leishmania* pathogenesis. The work with peritoneal macrophages proved itself a valid model since C3H/He and BALB/c cells, when infected by *L*. *amazonensis*, presented differences in infection rate and parasite load. Stimulation with Th1 or Th2 cytokines were able to modulate infection, altering infection rate, the amount of parasites per cell and production of NO and H_2_O_2_, as well as arginase activity. However, none of these mechanisms was able to explain the resistance of C3H/He macrophages.

The analysis of infection in BALB/c and C3H/He peritoneal macrophages showed no difference in the infection rate in the first 48 hours, suggesting that the genetic of the host does not exert influence in the initial time of infection. Nevertheless, after 72h of infection, peritoneal macrophages of C3H/He mice showed a lower percentage of infected cells and less proliferation of amastigotes than BALB/c macrophages. These results suggest that *L*. *amazonensis* amastigotes can proliferate on cells from both backgrounds, but C3H/He macrophages have mechanisms that can limit multiplication of the parasite or are able to maintain a stable infection, whereas BALB/c macrophages allow the increase of parasite load. These observations corroborate the *in vivo* experiment, in which BALB/c is susceptible to infection, developing a growing ulcer, while C3H/He develops only a small lesion which self-resolves [18,20]. An in vitro study from another group also demonstrated that BALB/c macrophages are susceptible to infection by *L*. *amazonensis*, but they observed a slight non-significant decrease in the infection rate, from 94.3% at 24h to 83.5% at 72h post infection. On the other hand, they observed an increase in the average number of amastigotes per cell [28]. In our work, we also observed an increase in the number of amastigotes per cell through time corroborating their data.

Once established that there was a difference in the multiplication of amastigotes in BALB/c and C3H/He macrophages, we departed to evaluate the mechanisms involved in parasite control or proliferation. The *L*. *major* infection model demonstrated the relevance of the Th1/Th2 model in vivo [25]. In general, this paradigm of resistance/susceptibility demonstrates that a protective response against *L*. *major* parasites is associated with a Th1-type cellular immune response with IFN-γ production, typically found in the C57BL/6 model; and a non-protective response is associated with a Th2-type cellular immune response with IL-4 production, usually seen in BALB/c mice [29]. This immune response can determine the type of macrophage activation, classic or alternative [9,12]. Although this paradigm holds true for *L*. *major*, in New World *Leishmania* species, including *L*. *amazonensis*, this dichotomy is not well established [30] and both BALB/c and C57BL/6 mouse are susceptible to *L*. *amazonensis* infection [20].

Accordingly, we challenged BALB/c and C3H/He macrophages with Th1 and Th2 stimuli in order to change the susceptibility profile and test its influence on parasite host resistance/susceptibility to *L*. *amazonensis* infection and NO production. In experiments without any stimulus, NO production was higher in C3H/He cells, which also presented lower infection rate and parasitic burden. When C3H/He and BALB/c macrophages were stimulated with LPS and IFN-γ, NO production increased and infection rate decreased on both cells. However, although C3H/He macrophages produced more NO than BALB/c cells, infection rate and parasite burden was equal on both cells. In non-stimulated macrophages infected with *L*. *major*, there was no difference in NO production between BALB/c (susceptible) and C57BL/6 (resistant) [31]. When they were stimulated with IFN-γ and LPS, the total parasite load decreased on both cells, but, surprisingly, BALB/c macrophages produced NO at a higher concentration than C57BL/6’s [31]. Some authors reported that macrophages derived from the bone marrow of C3HeB/FeJ mice infected with *L*. *amazonensis* or *L*. *major* promastigotes present similar infection rate 72 hours after infection. However, when cells are activated with LPS and IFN-γ, the number of *L*. *major*-infected cells declines, while no difference is observed on *L*. *amazonensis*-infected cultures, arguing that *L*. *amazonensis* parasites are not susceptible to NO killing [32]. Contrary to these results, we demonstrated that, similarly to *L*. *major*, *L*. *amazonensis* is also sensitive to the effect of NO, since the increase in its production causes a reduction in the infection rate. These results corroborate the role of Th1 stimuli in favoring parasite clearance, regardless of the genetic background of the host.

Concerning the arginase pathway, no difference in the enzyme activity was observed in the non-stimulated protocol, despite the fact that we observed differences in the parasite load and infection rate between BALB/c and C3H/He macrophages. However, in the protocol with Th2 stimulus it was possible to observe a difference in arginase activity among the macrophage strains, which was associated with a difference in the total parasite load. The same results were observed in mice with distinct susceptibility to *L*. *infantum* infection [33]. This confirms the idea that increased arginase activity favors parasite proliferation, regardless of the host susceptibility profile. IL-4 is known to modulate the host immune response, inhibiting NO production by macrophages and preventing its activation by the classical pathway [34].

To determine if NO was playing a role in limiting *L*. *amazonensis* infection *in vitro*, we inhibited iNOS activity using a specific inhibitor. 1400W was shown to strongly and selectively inhibit iNOS, without effect on the constitutive enzyme [24]. As we suppressed NO production, infection rate increased in both cells. Similarly, when Th1-activated macrophages from C3HeB/FeJ mice were infected with *L*. *major* or *L*. *amazonensis* and treated with LN- (1-iminoethyl) lysine, another iNOS-specific inhibitor, their ability to eliminate parasites was also abrogated, resulting in a significant increase in the number of infected macrophages compared to untreated activated macrophages [32]. Together, these findings demonstrate that iNOS plays an important role in limiting infection independent of the *Leishmania* or host species. Nevertheless, other authors argue that elimination of *L*. *infantum* amastigotes is dependent on the action of NO, whereas the control of *L*. *amazonensis* is independent [35]. In our study, the inhibition of iNOS resulted in a significant increase in the parasite load and infection rate. Nevertheless, C3H/He macrophages maintained lower parasite load than BALB/c cells, showing that the observed differences between the lineages could not be eliminated by iNOS inhibition. This result corroborates the idea that NO could act as a cytostatic agent against *L*. *amazonensis*, supporting the need for other factors, such as ROS, to kill the amastigotes [32,36]. It is know that NO alone is not sufficient to control infection and may contribute to the tissue damage observed in human cutaneous leishmaniasis [27]. This damaged tissue is observed in later times during *L*. *amazonensis* infection in BALB/c mice [20].

In order to study the contribution of ROS in parasite control, we evaluated the production of H_2_O_2_, to see whether it could explain the differences in infection observed between BALB/c and C3H/He macrophages. No difference was observed in the non-stimulated and Th2-stimulated protocols, but when cell received a Th1 stimulus, H_2_O_2_ production was increased and C3H/He cells produced significantly more H_2_O_2_ than BALB/c. This result corroborates the literature findings, showing that IFN-γ enhances the respiratory burst in macrophages, leading to ROS production and better parasite elimination [37]. It also supports the idea that *Leishmania* parasites are sensitive to ROS, but in non-activated macrophages the production is insufficient to eliminate the parasites [37].

Altogether our results show that both NO and H_2_O_2_ are involved in restraining *L*. *amazonensis* infection in macrophages. However, differences in the production of these molecules could not explain the differences in infection rate between BALB/c and C3H/He. Moreover, iNOS inhibition could not suppress interstrain differences. Therefore, we conclude that, in our model of infection, NO and H_2_O_2_, in spite of their role, are not the principal factors in the resistance to *L*. *amazonensis* infection. Whatever mechanisms make C3H/He macrophages more resistant to infection than BALB/c, it is still to be described.

Further studies are being carried out to find other factors that might be implicated in C3H/He resistance. The *Slc1a1* (formelly *Nramp1*) gene have been described to be associated with susceptibility to multiple pathogens, including *L*. *donovani* [38]. The gene encodes for a transmembrane transporter that carries divalent cations out of the phagossome innerspace, depriving parasites from these ions [39]. C3H/HeJ animals are described to have a resistant allele while BALB/cJ carries the susceptible one [40]. A recent systematic review and meta-analysis have found some SLC11A1 polymorphisms to be associated with susceptibility to both cutaneous and visceral human leishmaniasis [41]. Meanwhile, genome wide association studies in Brazilian and Indian populations have implicated only HLA genes in the susceptibility to leishmaniasis [42]. Either way, susceptibility to leishmaniasis is clearly a complex trait with a multifactorial etiology that needs further studies. Uncovering the molecular basis of host resistance could provide valuable tools in the search for a vaccine or more efficient treatment.

## Supporting information

S1 Fig Effect of Th2 or Th1 stimuli in BALB/c and C3H/He macrophage infection. Peritoneal macrophages of BALB/c and C3H/He were infected with *L*. *amazonensis* promastigotes (2 MOI). After Giemsa staining, 100 cells/coverslip were counted in order to estimate the percentage of infected cells, mean number of amastigotes per cell and total amount of amastigotes in a hundred cells at 24, 48, 72 and 96 hours after infection. (A-C) non-stimulated cells versus IL-4 (2 ng/mL) stimulated cells and (D-F) non-stimulated cells versus IFN-γ (2 ng/mL) and LPS (5 μg/mL) stimulated cells. Statistical analysis were performed by ANOVA followed by a Tukey’s multiple comparisons test. *p<*0*,*05*; **p<*0*,*01*, ***p<*0*,*001*, ****p<*0*,*0001*. Bars represent mean ± SD.
(TIF)

S2 Fig Nitric oxide production after iNOS inhibition. Peritoneal macrophages of BALB/c and C3H/He were treated with 100 μM of 1400W dihydrochloride after macrophage plating and infection with *L*. *amazonensis* (2 MOI). Nitrite production was measured by Griess reaction. No difference was observed between non-stimulated and iNOS-inhibited cells. Statistical analysis were performed by ANOVA followed by a Tukey’s multiple comparisons test. ****p<*0*,*0001*. Bars represent mean ± SD.
(TIF)

S1 Table Number of cells and type of plate used in the experiments. (PDF)

## References

[1] WHO WHO. Leishmaniasis [Internet]. 2019;

[2] Organização Pan-Americana da Saúde. Informe Epidemiológico das Américas. Leishmanioses. Informe de Leishmanioses No 5. 2017.:8.

[3] Basano S de A, Camargo LMA. Leishmaniose tegumentar americana: histórico, epidemiologia e perspectivas de controle. Vol. 7, Revista Brasileira de Epidemiologia. 2004 7(3):328–37.

[4] McGwire BS, Satoskar AR. Leishmaniasis: clinical syndromes and treatment. Vol. 107, QJM: monthly journal of the Association of Physicians. 2014 107(1):7–14. 10.1093/qjmed/hct116 23744570 PMC3869292

[5] Horta MF, Mendes BP, Roma EH, Noronha FSM, MacDo JP, Oliveira LS, et al Reactive oxygen species and nitric oxide in cutaneous leishmaniasis. Vol. 2012, Journal of Parasitology Research. 2012 2012. 10.1155/2012/203818 PMC3337613 22570765

[6] Gupta G, Oghumu S, Satoskar AR. Mechanisms of Immune Evasion in Leishmaniasis. Vol. 82, Advances in Applied Microbiology. 2013 82:155–84. 10.1016/B978-0-12-407679-2.00005-3 23415155 PMC3697132

[7] Scorza BM, Carvalho EM, Wilson ME. Cutaneous manifestations of human and murine leishmaniasis. Vol. 18, International Journal of Molecular Sciences. 2017 18(6). 10.3390/ijms18061296 PMC5486117 28629171

[8] Wanasen N, Soong L. L-arginine metabolism and its impact on host immunity against Leishmania infection. Vol. 41, Immunologic Research. 2008 41(1):15–25. 10.1007/s12026-007-8012-y 18040886 PMC2639710

[9] Liu D, Uzonna JE. The early interaction of Leishmania with macrophages and dendritic cells and its influence on the host immune response. Vol. 2, Frontiers in Cellular and Infection Microbiology. 2012 2(June):1–8. 10.3389/fcimb.2012.00001 22919674 PMC3417671

[10] Malyshev I, Malyshev Y. Current concept and update of the macrophage plasticity concept: Intracellular mechanisms of reprogramming and M3 macrophage “switch” phenotype. Vol. 2015, BioMed Research International. 2015 2015. 10.1155/2015/341308 PMC4561113 26366410

[11] Patel U, Rajasingh S, Samanta S, Cao T, Dawn B, Rajasingh J. Macrophage polarization in response to epigenetic modifiers during infection and inflammation. Vol. 22, Drug Discov Today. 2017 22(1):186–93. 10.1016/j.drudis.2016.08.006 27554801 PMC5226865

[12] Classen A, Lloberas J, Celada A. Macrophage Activation: Classical vs. Alternative In: Reiner NE, editor. Vol. 531, Macrophages and Dendritic Cells, Methods and Protocols. Humana Press; 2009; p. 29–43. 10.1007/978-1-59745-396-7_3 19347309

[13] Comalada M, Yeramian A, Modolell M, Lloberas J, Celada A. Arginine and Macrophage Activation In: Ashman RB, editor. Vol. 844, Leucocytes, Methods and Protocols,. Humana Press; 2012; p. 223–35. 10.1007/978-1-61779-527-5_16 22262446

[14] Muraille E, Leo O, Moser M. Th1/Th2 paradigm extended: Macrophage polarization as an unappreciated pathogen-driven escape mechanism? Vol. 5, Frontiers in Immunology. 2014 5(NOV):1–12. 10.3389/fimmu.2014.00001 25505468 PMC4244692

[15] França-Costa J, Van Weyenbergh J, Boaventura VS, Luz NF, Malta-Santos H, Oliveira MCS, et al Arginase I, polyamine, and prostaglandin E2pathways suppress the inflammatory response and contribute to diffuse cutaneous leishmaniasis. Vol. 211, Journal of Infectious Diseases. 2015 211(3):426–35. 10.1093/infdis/jiu455 25124926

[16] Mendes Wanderley JL, Costa JF, Borges VM, Barcinski M. Subversion of immunity by Leishmania amazonensis parasites: Possible role of phosphatidylserine as a main regulator. Vol. 2012, Journal of Parasitology Research. 2012 2012. 10.1155/2012/981686 PMC3306939 22518276

[17] Kropf P, Freudenberg N, Kalis C, Modolell M, Herath S, Galanos C, et al Infection of C57BL/10ScCr and C57BL/ 10ScNCr mice with Leishmania major reveals a role for Toll-like receptor 4 in the control of parasite replication. Vol. 76, Journal of Leukocyte Biology. 2004 76:48–57. 10.1189/jlb.1003484 15039466

[18] de Oliveira Cardoso F, de Souza C da SF, Mendes VG, Abreu‐Silva AL, Gonçalves da Costa SC, Calabrese K da S. Immunopathological Studies of *Leishmania amazonensis* Infection in Resistant and in Susceptible Mice. Vol. 201, The Journal of Infectious Diseases. 2010 201(12):1933–40. 10.1086/652870 20462353

[19] Silva-Almeida M, Carvalho LO, Abreu-Silva AL, Souza CS, Hardoim DJ, Calabrese KS. Extracellular matrix alterations in experimental Leishmania amazonensis infection in susceptible and resistant mice. Vol. 43, Veterinary Research. 2012 43(1):1–9. 22316002 10.1186/1297-9716-43-10 PMC3395857

[20] de Souza C, Calabrese K, Abreu-Silva A, Carvalho L, Cardoso F, Dorval M, et al Leishmania amazonensis isolated from human visceral leishmaniasis: histopathological analysis and parasitological burden in different inbred mice. Vol. 33, Histol Histopathol. 2018 33(7):705–16. 10.14670/HH-11-965 29345298

[21] Green LC, Wagner D a, Glogowski J, Skipper PL, Wishnok JS, Tannenbaum SR. Analysis of Nitrate, Nitrite, and [15N ] Nitrate in Biological Fluids Automated NO; and NO? Analysis. Vol. 126, Analysis. 1982126(1):131–8. 10.1016/0003-2697(82)90118-x 7181105

[22] Pick E, Keisari Y. A simple colorimetric method for the measurement of hydrogen peroxide produced by cells in culture. Vol. 38, Journal of Immunological Methods. 1980 38(1–2):161–70. 10.1016/0022-1759(80)90340-3 6778929

[23] Pick E, Mizel D. Rapid microassays for the measurement of superoxide and hydrogen peroxide production by macrophages in culture using an automatic enzyme immunoassay reader. Vol. 46, Journal of Immunological Methods. 1981 46(2):211–26. 10.1016/0022-1759(81)90138-1 6273471

[24] Garvey EP, Oplinger JA, Furfine ES, Kiff RJ, Laszlo F, Whittle BJR, et al 1400W is a slow, tight binding, and highly selective inhibitor of inducible nitric-oxide synthase in vitro and in vivo. Vol. 272, Journal of Biological Chemistry. 1997 272(8):4959–63. 10.1074/jbc.272.8.4959 9030556

[25] Sacks D, Noben-Trauth N. The immunology of susceptibility and resistance to Leishmania major in mice. Vol. 2, Nature Reviews Immunology. 2002 2(11):845–58. 10.1038/nri933 12415308

[26] Ganguli G, Mukherjee U, Sonawane A. Peroxisomes and Oxidative Stress: Their Implications in the Modulation of Cellular Immunity During Mycobacterial Infection. Vol. 10, Frontiers in Microbiology. 2019 10(June):1–17. 31258517 10.3389/fmicb.2019.01121 PMC6587667

[27] Carneiro PP, Conceição J, Macedo M, Magalhães V, Carvalho EM, Bacellar O. The role of nitric oxide and reactive oxygen species in the killing of Leishmania braziliensis by monocytes from patients with cutaneous leishmaniasis. Vol. 11, PLoS ONE. 2016 11(2):1–16. 10.1371/journal.pone.0148084 PMC4739692 26840253

[28] Ramos PKS, Brito MDV, Silveira FT, Salgado CG, De Souza W, Picanço-Diniz CW, et al In vitro cytokines profile and ultrastructural changes of microglia and macrophages following interaction with Leishmania. Vol. 141, Parasitology. 2014 141(8):1052–63. 10.1017/S0031182014000274 24717447

[29] Hurdayal R, Brombacher F. Interleukin-4 receptor alpha: From innate to adaptive immunity in murine models of cutaneous leishmaniasis. Vol. 8, Frontiers in Immunology. 2017 8(NOV). 10.3389/fimmu.2017.01354 PMC5686050 29176972

[30] McMahon-Pratt D, Alexander J. Does the Leishmania major paradigm of pathogenesis and protection hold for New World cutaneous leishmaniases or the visceral disease? Vol. 201, Immunological Reviews. 2004 201:206–24. 10.1111/j.0105-2896.2004.00190.x 15361243

[31] Sans-Fons MG, Yeramian A, Pereira-Lopes S, Santamaría-Babi LF, Modolell M, Lloberas J, et al Arginine transport is impaired in C57Bl/6 mouse macrophages as a result of a deletion in the promoter of Slc7a2 (CAT2), and susceptibility to Leishmania infection is reduced. Vol. 207, Journal of Infectious Diseases. 2013 207(11):1684–93. 10.1093/infdis/jit084 23460752

[32] Mukbel R, Patten CJ, Gibson K, Ghosh M, Petersen C, Jones D. Macrophage Killing of Leishmania Amazonensis Amastigotes Requires Both Nitric Oxide and Superoxide. Vol. 76, The American Journal of Tropical Medicine and Hygiene. 2007 76(4):669–75. 17426168

[33] Iniesta V, Gómez-Nieto LC, Corraliza I. The Inhibition of Arginase by N ω -Hydroxy-l-Arginine Controls the Growth of Leishmania Inside Macrophages. Vol. 193, The Journal of Experimental Medicine. 2001 193(6):777–84. 10.1084/jem.193.6.777 11257143 PMC2193414

[34] Muxel SM, Aoki JI, Fernandes JCR, Laranjeira-Silva MF, Zampieri RA, Acuña SM, et al Arginine and polyamines fate in leishmania infection. Vol. 8, Frontiers in Microbiology. 2018 8(JAN):1–15. 10.3389/fmicb.2017.02682 PMC5775291 29379478

[35] de Souza Carmo ÉV, Katz S, Barbiéri CL. Neutrophils reduce the parasite burden in Leishmania (Leishmania) amazonensis-infected macrophages. Vol. 5, PLoS ONE. 2010 5(11). 10.1371/journal.pone.0013815 PMC2972777 21082032

[36] Gomes IN, De Carvalho Calabrich AF, Da Silva Tavares R, Wietzerbin J, Rodrigues De Freitas LA, Tavares Veras PS. Differential properties of CBA/J mononuclear phagocytes recovered from an inflammatory site and probed with two different species of Leishmania. Vol. 5, Microbes and Infection. 2003 5(4):251–60. 10.1016/s1286-4579(03)00025-x 12706438

[37] Scott P, Novais FO. Cutaneous leishmaniasis: Immune responses in protection and pathogenesis. Vol. 16, Nature Reviews Immunology. 2016 16(9):581–92. 10.1038/nri.2016.72 27424773

[38] Vidal S, Tremblay ML, Govoni G, Gauthier S, Sebastiani G, Malo D, et al The ity/lsh/bcg locus: Natural resistance to infection with intracellular parasites is abrogated by disruption of the nrampl gene. Vol. 182, Journal of Experimental Medicine. 1995 182(3):655–66. 10.1084/jem.182.3.655 7650477 PMC2192162

[39] Blackwell JM, Searle S, Mohamed H, White JK. Divalent cation transport and susceptibility to infectious and autoimmune disease: Continuation of the Ity/Lsh/Bcg/Nramp1/Slc11a1 gene story. Vol. 85, Immunology Letters. 2003 85(2):197–203. 10.1016/s0165-2478(02)00231-6 12527228

[40] Vidal SM, Malo D, Vogan K, Skamene E, Gros P. Natural resistance to infection with intracellular parasites: Isolation of a candidate for Bcg. Vol. 73, Cell. 1993 73(3):469–85. 10.1016/0092-8674(93)90135-d 8490962

[41] Braliou GG, Kontou PI, Boleti H, Bagos PG. Susceptibility to leishmaniasis is affected by host SLC11A1 gene polymorphisms: a systematic review and meta-analysis. Parasitology Research. 2019.(Desjeux 1996). 10.1007/s00436-019-06374-y 31230160

[42] Consortium L, 2 WTCCC, Fakiola M, Strange A, Cordell HJ, Miller EN, et al Common variants in the HLA-DRB1–HLA-DQA1 HLA class II region are associated with susceptibility to visceral leishmaniasis. Vol. 45, Nature Genetics. 2013 45:208 10.1038/ng.2518 23291585 PMC3664012

