# Supplementary figures and images for: Leishmania amazonensis resistance in murine macrophages: Analysis of possible mechanisms
Source: PLoS One. 2019 Dec 19;14(12):e0226837. doi: 10.1371/journal.pone.0226837 (PMC6922422; doi:10.1371/journal.pone.0226837)

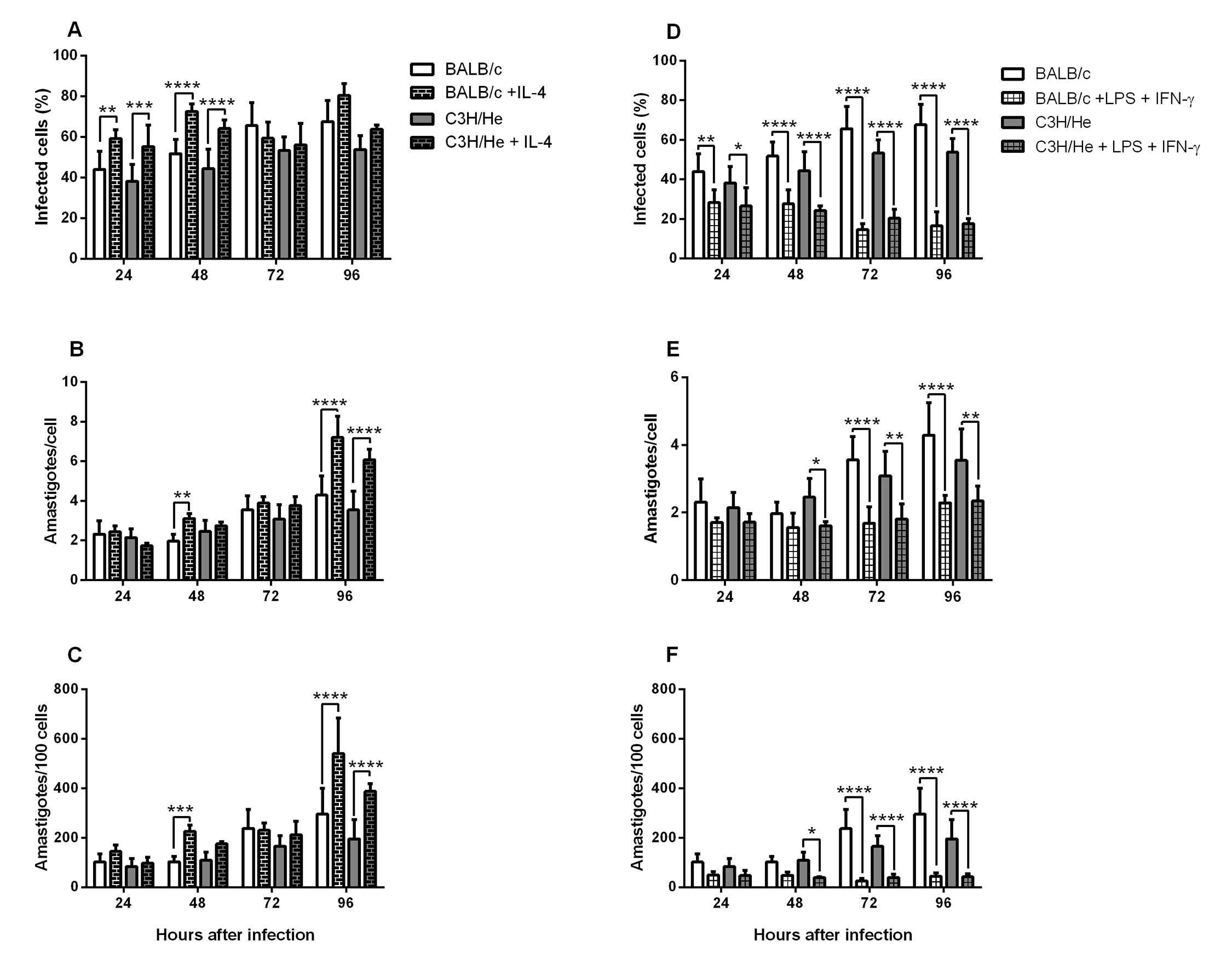

Supplement: S1 Fig — Peritoneal macrophages of BALB/c and C3H/He were infected with L. amazonensis promastigotes (2 MOI). After Giemsa staining, 100 cells/coverslip were counted in order to estimate the percentage of infected cells, mean number of amastigotes per cell and total amount of amastigotes in a hundred cells at 24, 48, 72 and 96 hours after infection. (A-C) non-stimulated cells versus IL-4 (2 ng/mL) stimulated cells and (D-F) non-stimulated cells versus IFN-γ (2 ng/mL) and LPS (5 μg/mL) stimulated cells. Statistical analysis were performed by ANOVA followed by a Tukey’s multiple comparisons test. *p<0,05; **p<0,01, ***p<0,001, ****p<0,0001. Bars represent mean ± SD. (TIF) [file pone.0226837.s001.tif]

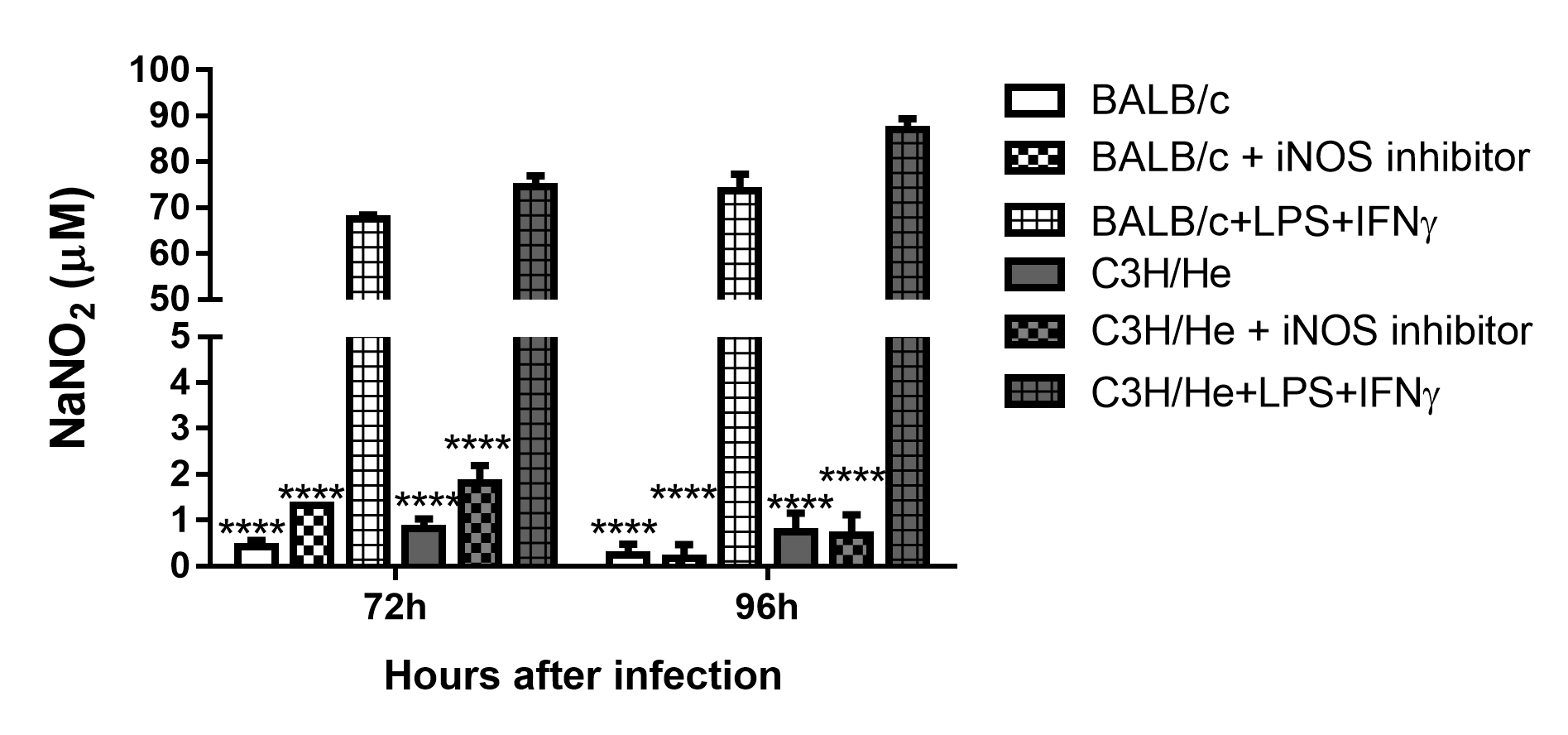

Supplement: S2 Fig — Peritoneal macrophages of BALB/c and C3H/He were treated with 100 μM of 1400W dihydrochloride after macrophage plating and infection with L. amazonensis (2 MOI). Nitrite production was measured by Griess reaction. No difference was observed between non-stimulated and iNOS-inhibited cells. Statistical analysis were performed by ANOVA followed by a Tukey’s multiple comparisons test. ****p<0,0001. Bars represent mean ± SD. (TIF) [file pone.0226837.s002.tif]
