# Supplementary material for: Leishmania amazonensis resistance in murine macrophages: Analysis of possible mechanisms
Source: PLoS One. 2019 Dec 19;14(12):e0226837. doi: 10.1371/journal.pone.0226837 (PMC6922422; doi:10.1371/journal.pone.0226837)
Supplement: S1 Table — (PDF) [file pone.0226837.s003.pdf]

**Experiment**	**Number of cells**	**Plate**
**Intracellular parasite load/ Inhibition of iNOS**	**$2x10^5$**	**24 wells**
**Arginase activity**	**$1x10^6$**	**96 wells**
**Nitric oxide production**	**$5x10^6$**	**24 wells**
**Hydrogen peroxide production**	**$1x10^6$**	**96 wells**
